# A computational approach for identification of core modules from a co-expression network and GWAS data

**DOI:** 10.1016/j.xpro.2021.100768

**Published:** 2021-08-21

**Authors:** Olivia L. Sabik, Cheryl L. Ackert-Bicknell, Charles R. Farber

**Affiliations:** 1 Center for Public Health Genomics, School of Medicine, University of Virginia, Charlottesville, VA 22908 USA; 2 Department of Biochemistry and Molecular Genetics, School of Medicine, University of Virginia, Charlottesville, VA 22908 USA; 3 Department of Orthopedics, Anschutz Medical Campus, University of Colorado, Aurora, CO, USA; 4 Department of Public Health Sciences, University of Virginia, Charlottesville, VA 22908, USA

**Keywords:** Bioinformatics, Genetics, Genomics, RNAseq, Systems biology

## Abstract

This protocol describes the application of the “omnigenic” model of the genetic architecture of complex traits to identify novel “core” genes influencing a disease-associated phenotype. Core genes are hypothesized to directly regulate disease and may serve as therapeutic targets. This protocol leverages GWAS data, a co-expression network, and publicly available data, including the GTEx database and the International Mouse Phenotyping Consortium Database, to identify modules enriched for genes with “core-like” characteristics.

For complete details on the use and execution of this protocol, please refer to Sabik et al. (2020).

## Highlights

• A protocol to identify core modules by integrating gene expression data and GWAS data
• Core modules serve as the basis for identifying genes influencing complex traits
• Disease gene identification by module integration with publicly available databases

## Graphical abstract

**Figure:**
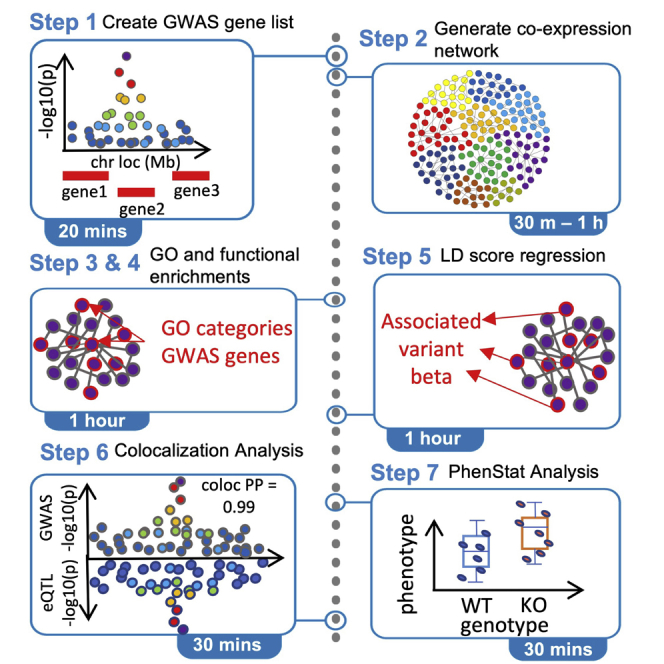

## Before you begin

### Download the vignette demonstrating the protocol

**Timing: 0.25 h**

1. An R markdown notebook and html markdown document walking through the steps described in this protocol are available on github at https://github.com/Farber-Lab/STAR_protocols_core_modules.
2. From this github repository, either download the file star_protocols_vignette.html and open it in your browser to view the code and output, or clone the repository, open star_protocols_vignette.Rmd in RStudio to run the code locally. If you clone the repo and run the code locally, set your working directory accordingly and all of the paths will resolve.

### Identify a GWAS study with available summary statistics related to your trait of interest

**Timing: 0.5–1 h**

3. Search the GWAS catalog for a study with summary statistics related to your trait of interest or use the results of an internally run GWAS study

This protocols requires GWAS summary statistics as an input. Users can either bring their own GWAS study, or search the GWAS catalog. GWAS catalog contains 1376 published studies with available summary statistics across hundreds of phenotypes (Buniello et al., 2019). You can search the catalog in order to identify a study with sufficient data to support the downstream analyses in this protocol, namely that the summary statistics are available. It is possible to execute portions of this protocol without full summary statistics; however, you will not be able to use colocalization methods to relate the GWAS results to expression quantitative trait loci data (eQTL). While there is no strict threshold for the size of the GWAS study to use in this analysis, it will be difficult to identify enriched co-expression modules from a small set of genes. GWAS studies should identify on the order of 10s–100s of significant loci.

### Acquire RNA-seq data in the relevant tissue or cell type for your trait of interest

**Timing: 1 day to 8 weeks (depending on availability of data)**

4. Identify either an existing RNA-seq data or a co-expression network from a relevant cell type or tissue for your trait of interest.

The basis of this protocol is a co-expression network representing the functional co-expression of genes within the tissue or cell-type relevant to your trait of interest. There may be a publicly available source of RNA-seq data or a previously published co-expression network that is suitable for your applications. Search the literature or the Gene Expression Omnibus (Barrett et al., 2012; Edgar et al., 2002) to identify sources of data that can support the study. However, it is important that the expression data used in this protocol is from a tissue or cell type that is relevant to your trait of interest, otherwise the GWAS signals will not align with the functional organization of the genes in the network.

***Note:*** It is important that the co-expression network is the result of an expression study that had a large enough sample size. WGCNA, a popular package for constructing co-expression networks recommends at least 15, but ideally, 20 or more samples (Langfelder and Horvath, 2008).

5. If no publicly available data exists, identify a source of relevant cells and tissue from which you can extract RNA and perform RNA-seq. This data could come from human or mouse primary cells or a relevant cell line, however, the cell type or tissue must be relevant to the trait of interest. For an example, see our study of genes influencing bone mineral density using murine osteoblasts (Sabik et al., 2020).

***Note:*** The protocol for processing and extracting RNA will be specific to your cell type or tissue, and this part of the process will not be covered in this protocol.

### Pre-processing RNA-seq data for co-expression network construction

**Timing: 2 h**

6. The main section of this protocol assumes you have an expression matrix that is pre-processed and ready for input to WGCNA (Langfelder and Horvath, 2008). Information about processing RNA-seq data can be found in the FAQ section of the WGCNA package. Briefly, starting from raw count data, it is recommended that genes with low expression across the majority of samples are filtered out, e.g., all features with fewer than 10 counts in 90% of the samples, and a variance-stabilizing transformation (DESeq2 package) is performed (Love et al., 2014). Normalized counts (RPKM/FPKM) can also be log transformed (log2(x+1)) and used to construct the network. The result of this process is a matrix of expression values (rows) by samples (columns).

>library(DESeq2)

>DESeq2::varianceStabilizingTransformation(object = exp_mat)

or

>log2(exp_mat + 1)

***Optional:*** An optional step is to remove batch effects from the expression data using PEER (Stegle et al., 2012). PEER can be used to remove both known and latent batch effects from data. The developers of PEER have produced detailed tutorials, available on their Github wiki page.

7. Finally, a quantile normalization is performed to reduce experimental noise from the expression matrix (Bolstad, 2020).

>library(preprocessCore)

>norm_exp_mat = preprocessCore::normalize.quantiles(as.matrix(exp_mat))

>colnames(norm_exp_mat) = colnames(exp_mat)

>rownames(norm_exp_mat) = rownames(exp_mat)

>norm_exp_mat = as.data.fram(norm_exp__mat)

### Curating lists of known disease and phenotype-associated genes

**Timing: 2–5 days**

8. One characteristic of core genes is that they are associated with monogenic diseases related to your trait of interest (Boyle et al., 2017). For example, given an interest in bone mineral density GWAS, one would curate a list of genes that cause monogenic bone diseases (Sabik et al., 2020).
  a. Thus, this step includes identifying a specific set of monogenic or Mendelian diseases that are related to your trait of interest
  b. Then, continue with a literature search to curate a list of genes experimentally shown to cause these diseases
    i. For this purpose, you may want to consult the Online Mendelian Inheritance in Man (OMIM) database (McKusick, 2007) or eDGAR (Babbi et al., 2017)
9. Another characteristic of core genes is that they are strongly associated with abnormal phenotypes in knockout models (Boyle et al., 2017), and so another resource that can be used to identify core modules is a curated list of genes which produce a phenotype related to your trait of interest when knocked out.
  a. There are databases of gene perturbations that result in a variety of phenotypes, including the Mouse Genome Informatics database (MGI) and the International Mouse Phenotyping Consortium (IMPC) (Bolser, 2004; Koscielny et al., 2014).
    i. The IMPC database systematically knocks out genes and conducts a whole panel of phenotyping relating to various organ systems, development, neurology and behavior, aging and mortality, metabolism, reproduction, etc.
    ii. The MGI database is a systematic repository for observed phenotypes in experimental systems. Using their mammalian phenotype browser, search for phenotypes related to your trait of interest and create a list of relevant phenotypes and their associated genes.
    iii. These are just two examples of data repositories that contain associations between traits or diseases and genes. Be sure to do some research to see if your specific phenotype has a more targeted repository. For example, the Bonebase Database has deep bone phenotyping for many of the knockout mice produced in the knockout mouse project (KOMP) (Rowe et al., 2018).
10. Save all of the genes from this section comma separated value (csv) files for use during the protocol.
11. While there is no strict threshold for how large these lists of genes should be, the enrichment analysis will not identify any significantly enriched modules in Step 4, module enrichment.

## Key resources table

**Table:** 

REAGENT or RESOURCE	SOURCE	IDENTIFIER
**Deposited data**		

Collaborative cross RNA sequencing	Sabik et al., 2020	GEO: GSE134081

**Software and algorithms**		

R	(R Core Team, 2020)	https://www.r-project.org/
RStudio	(R Studio Team, 2020)	https://rstudio.com/
Python	(Van Rossum and Others, 2007)	https://www.python.org/
Tidyverse	(Wickham et al., 2019)	https://www.tidyverse.org/
Knitr	(Xie, 2014)	https://yihui.org/knitr/
BiocManager	(Morgan, 2018)	https://cran.r-project.org/web/packages/BiocManager/index.html
biomaRt	(Durinck et al., 2009)	https://bioconductor.org/packages/release/bioc/html/biomaRt.html
Devtools	(Wickham and Chang, 2016)	https://cran.r-project.org/web/packages/devtools/index.html
GTExIdConverter	NA	https://github.com/oliviasabik/GTExIdConverter
DESeq2	(Love et al., 2014)	http://bioconductor.org/packages/release/bioc/html/DESeq2.html
PEER	(Stegle et al., 2012)	https://github.com/PMBio/peer/wiki
WGCNA	(Langfelder and Horvath, 2008)	https://cran.r-project.org/web/packages/WGCNA/index.html
ToppFun	(Chen et al., 2009)	https://toppgene.cchmc.org/
LDlinkR	(Machiela and Chanock, 2015)	https://ldlink.nci.nih.gov/?tab=home
GenomicRanges	(Lawrence et al., 2013)	https://bioconductor.org/packages/release/bioc/html/GenomicRanges.html
LDSC	(Finucane et al., 2015)	https://github.com/bulik/ldsc
Coloc	(Giambartolomei et al., 2014)	https://cran.r-project.org/web/packages/coloc/index.html
RACER	(Sabik and Farber, 2018)	https://github.com/oliviasabik/RACER
PhenStat	(Kurbatova et al., 2015)	https://www.bioconductor.org/packages/release/bioc/html/PhenStat.html

## Materials and equipment

Data (expression matrix, pre-processed and ready for WGCNA--see before you begin). The data listed in the key resources tables can be used as exemplar expression data.

R software and required packages. This protocol describes a computational approach that is based on numerous packages available in R. While newer versions of some of these packages exist, this protocol was developed with R v3.4.2, RStudio v1.1.383, and the indicated package versions below:

• tidyverse (v1.2.1)
• knitr (v1.24)
• BiocManager (v1.30.10)
• DESeq2 (v1.18.1)
• WGCNA (v1.68)
• biomaRt (v2.34.2)
• LDLinkR (v1.0.2)
• Genomic Ranges (v1.30.3)
• coloc (v3.2-1)
• RACER (v1.0.0)
• PhenStat (v2.14.0)
• devtools (2.0.2)
• GTExIdConverter (0.0.0.9000)

Optionally, the python (v2.7.15) packages LDSC (v1.0.0) and PEER (v1.3)

Local--Memory: 8GB required, 16GB recommended; Processors: 1 required, 4 recommended

Remote--Memory: 200 GB or more; Processors: 1 required

## Step-by-step method details

***Note:*** Further discussion of the research questions underlying this protocol can be found in our two prior publications that leveraged this approach (Calabrese et al., 2016, Sabik et al., 2020). For example, in these publications, we explore the specificity of the module enrichments against other GWAS studies and more deeply characterize the topology of the genes in the annotated gene lists within the co-expression modules.

### Step 1: Creating GWAS gene list

**Timing: 20 min**

In order to identify co-expression modules enriched for GWAS genes, we first identify all genes overlapping a GWAS locus, defined as the set of SNPs in high linkage disequilibrium with the lead SNP.

1. Using the previously identified GWAS study, read in the list of lead SNPs that are significantly associated with the study trait. In many cases, this will be the output of fine-mapping (Schaid, et. al. 2018) or a file containing just the lead SNPs for each independent association. For downstream analysis, generate a table with these lead SNPs and their genome coordinates.
2. In order to programmatically access the LDLink data using the LDLinkR package in R, users must register on the LDLink website in order to receive an LDLink token. The token will be emailed to you.
3. Using this table and the LDLinkR package, each lead variant is queried in the 1000 Genomes database and the variants in linkage disequilibrium, or proxy SNPs, for each lead SNP are downloaded (1000 Genomes Project Consortium et al., 2012; Machiela and Chanock, 2015). In using this resource, you need to be aware of the population used in the GWAS study you are analyzing. In many cases, the European (“EUR”) population is representative of the population used in the study, however, this is not always the case. Compare the available populations from 1000 Genomes against the GWAS study publication to find the appropriate option for the analysis. Using the LDproxy_batch function, all GWAS SNPs can be queried at once, and the proxy SNPs will all be written to a file on disk using the append = TRUE option.

>LDproxy_batch(

 snp = bmd_snps$SNP,

 pop = “EUR”,

 r2d = “r2”,

 token = [insert_personal_token],

 append = true)

4. Next, this set of proxy SNPs is filtered to include only those with linkage disequilibirum R^2^ >= 0.7 with the lead variant. These are the regions of the genome that will determine which genes are implicated by the GWAS in cis.

>ld_regions = proxies %>%

 filter(r2 >= 0.7) %>%

 separate(col = coord, sep = “:”,

 into = c(“chr”, “coord”)) %>%

 group_by(query_snp %>%

 summarize(chr = max(chr),

 min = as.numeric(min(coord),

 max = as.numeric(max(coord)))

5. Then, using the Genomic Ranges tool, identify all genes from the GRCh37/hg19 Ensembl gene set overlapping a GWAS bin (Lawrence et al., 2013). Depending on the build of the genome used in the GWAS in the study, the correct gene annotation reference will need to be used. Download the reference file from the UCSC table browser (Haeussler et al., 2019).
  a. Create a GenomicRanges representation of both the GWAS regions and the reference gene set.
    >gr_genes = Granges(genes)
    >gr_gwas = Granges(ld07_gwas_regions)
  b. Next, identify the overlapping genes for each region using the findOverlaps function
    >overlaps = GenomicRanges::findOverlaps(gr_genes, gr_gwas)
    >overlaps_info = cbind(ld07_gwas_regions[overlaps@to,
     genes[overlaps@from,]$name)
    >overlaps_info = overlaps_info[,c(1,4,5)]
    >overlaps_info$type = “overlaps”
    >overlaps_info = overlaps_info[!duplicated(overlaps_info,]
    >colnames(overlaps_info) = c(“chromosome”, “gwas_snps”,
     “gene_name”, “type”)
  c. If no gene intersected a given region, include the nearest upstream and downstream genes from the region. These are identified using the “precede” and “follow” functions from GenomicRanges. The results are converted into data frames and filtered to include only nearest upstream and downstream genes associated with variants that do not overlap a gene.
    >nearest_precede = GenomicRanges::precede(gr_gwas,gr_genes)
    >precede_info = cbind(ld07_gwas_regions,
     genes[nearest_precede,])
    >precede_info = precede_info[,c(1,4,9)]
    >precede_info$type = “precede”
    >precede_info = precede_info[!duplicated(precede_info),]
    >colnames(precede_info) = c(“chromosome”, “gwas_snp”,
     “gene_name, “type”)
    >precede_info = precede_info[which(precede_info$morris_snp %in% overlaps_info$gwas_snp),]
    >nearest_follow = GenomicRanges::follow(gr_gwas,gr_genes)
    >follow_info = cbind(ld07_gwas_regions,
     genes[nearest_follow,])
    >follow_info = follow_info[,c(1,4,9)]
    >follow_info$type = “follow”
    >follow_info = follow_info[!duplicated(follow_info),]
    >colnames(follow_info) = c(“chromosome”, “gwas_snp”,
     “gene_name, “type”)
    >follow_info = follow_info[which(follow_info$morris_snp %in% overlaps_info$gwas_snp),]
  d. Combining these three results, create a table where each row represents a gene overlapping a region implicated by a GWAS-associated variant, the lead variant in that region, the chromosome, and whether the gene overlaps the region or is an upstream/downstream gene.

>gwas_region_genes = rbind(overlaps_info,

 precede_info, follow_info)

6. Finally, the list of human genes is converted to mouse homologs using the MGI mouse to human homology map (Bolser, 2004) (http://www.informatics.jax.org/downloads/reports/HOM_MouseHumanSequence.rpt).

>gwas_gene_hom = homology %>%

 filter(Symbol %in% gwas_region_genes$gene_name)

>gwas_mouse_hom = homology %>%

 filter(‘HomoloGene ID’ %in% gwas_gene_hom$’HomoloGene ID′) %>%

filter(‘NCBI Taxon ID’ == 10090)

### Step 2: Construct co-expression network

**Timing: 30 min to 1 h**

**Figure 1:**
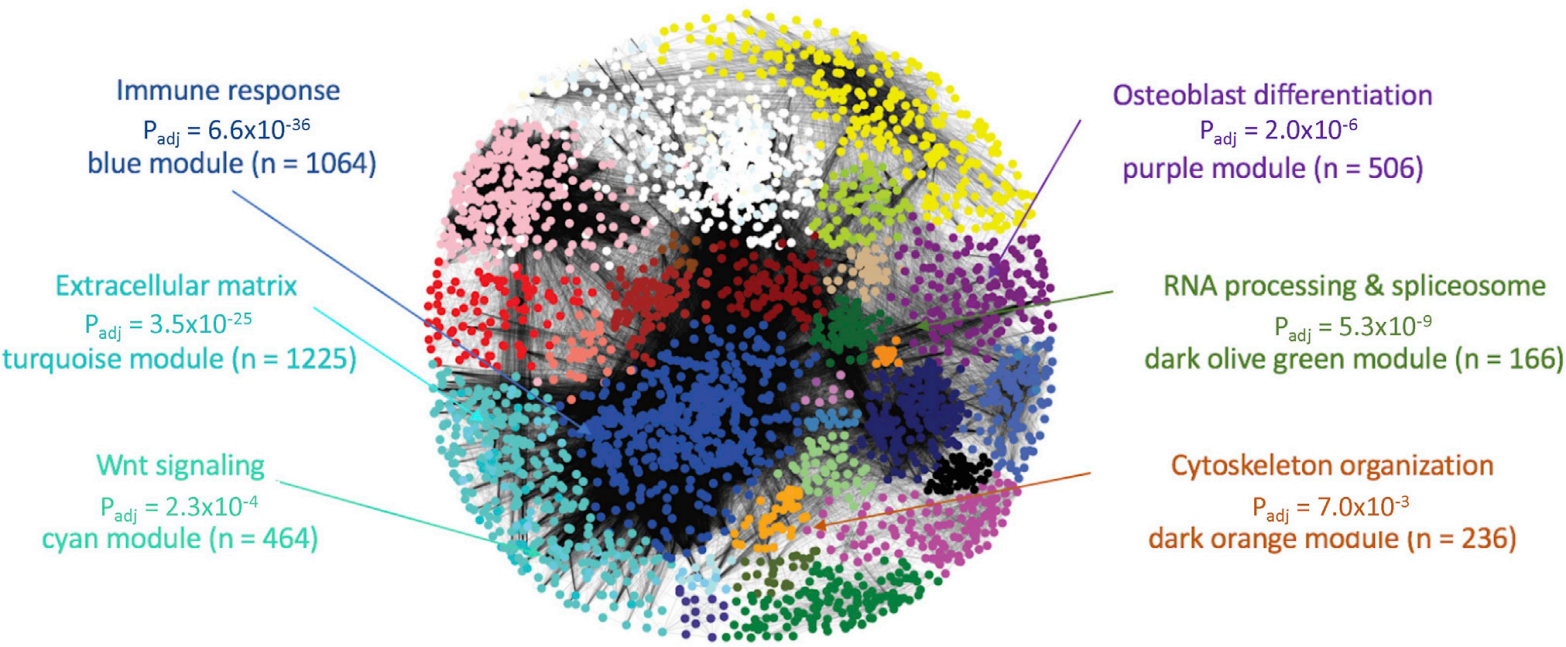
Example co-expression network from murine osteoblasts and GO annotations for individual modules As expected, some modules have generic functions, for example immune response and RNA processing, and others represent more specific functions, like osteoblast differentiation or Wnt signaling. Adapted from Figure 1 in (Sabik et al., 2020).

This step uses the WGCNA package to create a co-expression network (Figure 1) by grouping genes that co-vary across samples into modules (Langfelder and Horvath, 2008). This co-expression network serves as a basis for the enrichment analysis in the next set of steps. This section closely follows the WGCNA tutorials created by the authors of WGCNA, found here: https://horvath.genetics.ucla.edu/html/CoexpressionNetwork/Rpackages/WGCNA/Tutorials/.

7. Check the relationship among your samples using clustering to identify outliers.
  >sampleTree = hclust(dis(norm_exp_mat), method = “average”)
  a. Clustering and plotting the resulting tree will allow visualization of the relationships among your samples.
  b. As a result, you may want to remove samples that are dramatically different from the mean by cutting the tree using cutHeight; more details can be found in the WGCNA data processing tutorial.

8. Next, the soft-thresholding power is empirically determined using the pickSoftThreshold function. Because it is assumed that the co-expression network will have a scale-free topology, the scale-free topology fit index is calculated across a range of powers, and the minimum power required to reach the point of diminishing returns is selected as the soft thresholding power. This power is applied to the adjacency matrix prior to the calculation of the Topological Overlap Matrix.

>powers = c(c(1:10), seq(from = 12, to = 20, by = 2))

>sft = pickSoftThreshold(norm_exp_mat,

 powerVector = powers,

verbose = 5, networkType = “signed”)

***Note:*** This step may take a few minutes to run.

9. After determining the optimal soft-thresholding power, we can now calculate co-expression modules from the gene expression dataset. There are many parameters that can be tweaked in this function that can alter the resulting network. In this example, values close to the defaults, however the default values may not be optimal for your dataset. In this example, we used a minModuleSize of 20 genes and a mergeCutHeight of 0.15, in order to avoid having a large number of small modules. Additionally, a signed network was constructed, as Peter Langfelder recommends for biological network (Langfelder, 2018). For more information about the parameters of the network calculation step, consult the WGCNA help files in the R environment.

>net = blockwiseModules(norm_exp_mat, power = 8,

 TOMType = “signed”, minModuleSize = 20,

 networkType = “signed”, reassignThreshold = 0,

 mergeCutHeight = 0.15, numericLabels = TRUE,

 pamRespectsDendro = FALSE, saveTOMs = TRUE,

 saveTOMFileBase = “,/network/norm_exp_mat_TOM”,

 verbose = 3)

***Note:*** This step may take a while, from 15–20 min to run.

10. While the network is constructed, the topological overlap matrices (TOMs) are saved, however, we also suggest saving the soft-thresholding results, the tree cutting results, the module labels, colors, and eigengenes so they can be easily reloaded later without re-running any of the above code.

>moduleLabels = net$colors

>moduleColors = labels2colors(net$colors)

>MEs = net$MEs

>geneTree = net$dendrogram[[1]];

>save(sft, MEs, moduleLabels, moduleColors, geneTree,

 File = “./network/network.RData

**Pause point:** This is a good stopping point, as you can always come back and reload the network.RData file to come right back to where you are right now. To reload the network, use this code:

>load(“./data/network/network/RData”)

11. Finally, network results are all labeled with the identifiers from the expression matrix, however, it would also be useful to have them labeled with alternative identifiers that map to other applications we intend to use, including gene ontology analysis software and enrichment analysis with our curated gene lists. A common identifier for both of these applications in our case is gene symbol, however, this code can be modified for other organisms and ontologies.

>ensembl = useMart(“ensembl”)

>mm19 = useDataset(mart = ensembl, dataset = “mmusculus_gene_ensembl”)

>m = getBM(attributes = c(“ensemble_transcript_id”,

“external_gene_name”, “description”, “chromosome_name”,

“start_position”, “end_position”),

 filters = c(“ensembl_transcript_id”),

 values = colnames(norm_exp_mat),

 mart = mm19)

>gene_MEs = t(rbind(colnames(norm_exp_mat), moduleColors, moduleLabels))

>colnames(gene_MEs)[1:3] = c(“ensemble_transcript_id”,

“mod_color”, “mod_number”)

>annotated_mods = merge(gene_MEs, m, by =

“ensembl_transcript_id”)

### Step 3: Gene ontology analysis

**Timing: 30 min**

Next, perform gene ontology analysis for each module in the network to identify the functional categories represented by each module (Figure 1). There are numerous tools for conducting gene ontology analysis; however, our tool of preference for this project was ToppFun within the ToppGene suite (Chen et al., 2009). This web interface tool searches for enrichment across an extensive set of functional categories, including gene ontology and pathways, but also human and mouse phenotypes, publications and published co-expression data sets, gene families, microRNAs, drugs, and diseases. The background geneset used in ToppFun is the full set of genes present in the gene ontology categories that are being analyzed for enrichment.

**Pause point:** From this point on, each step break can be considered a good stopping point, just be sure to save your script.

12. In order to port our modules into the web interface and for ease of browsing, write each module out into a .csv file. For example, this code chunk writes out the genes in module 1 to a file that can be uploaded to the web interface.

>annotated_mods %>%

 filter(mod_number == 1) %>%

 write_csv(., “./data/network/mod_1.csv”)

13. Using the column of identifiers from this table, copy and paste into the ToppFun input box and begin the analysis. For each module of genes, ToppFun will report back a summary of all the enriched gene sets. Click the button at the top of the results page reading “Download All”, and save the results for browsing in R. ToppFun reports back the significance of each enrichment with an assortment of multiple testing correction methods, the number of hits for that category from your query ("Hit Count in Query List"), and the total number of genes in the category ("Hit Count in Genome"). ToppFun returns three different adjusted p-values: the Bonferroni, Benjamini & Hochberg, and Benjamini & Yekutieli adjusted values. We recommend using a threshold of Bonferroni corrected p-values less than 0.05 to identify significant enrichments. This is the most conservative approach, but given the large number of gene ontology categories, we have deemed the conservative approach appropriate.

### Step 4: Module enrichment

**Timing: 20 min**

**Figure 2:**
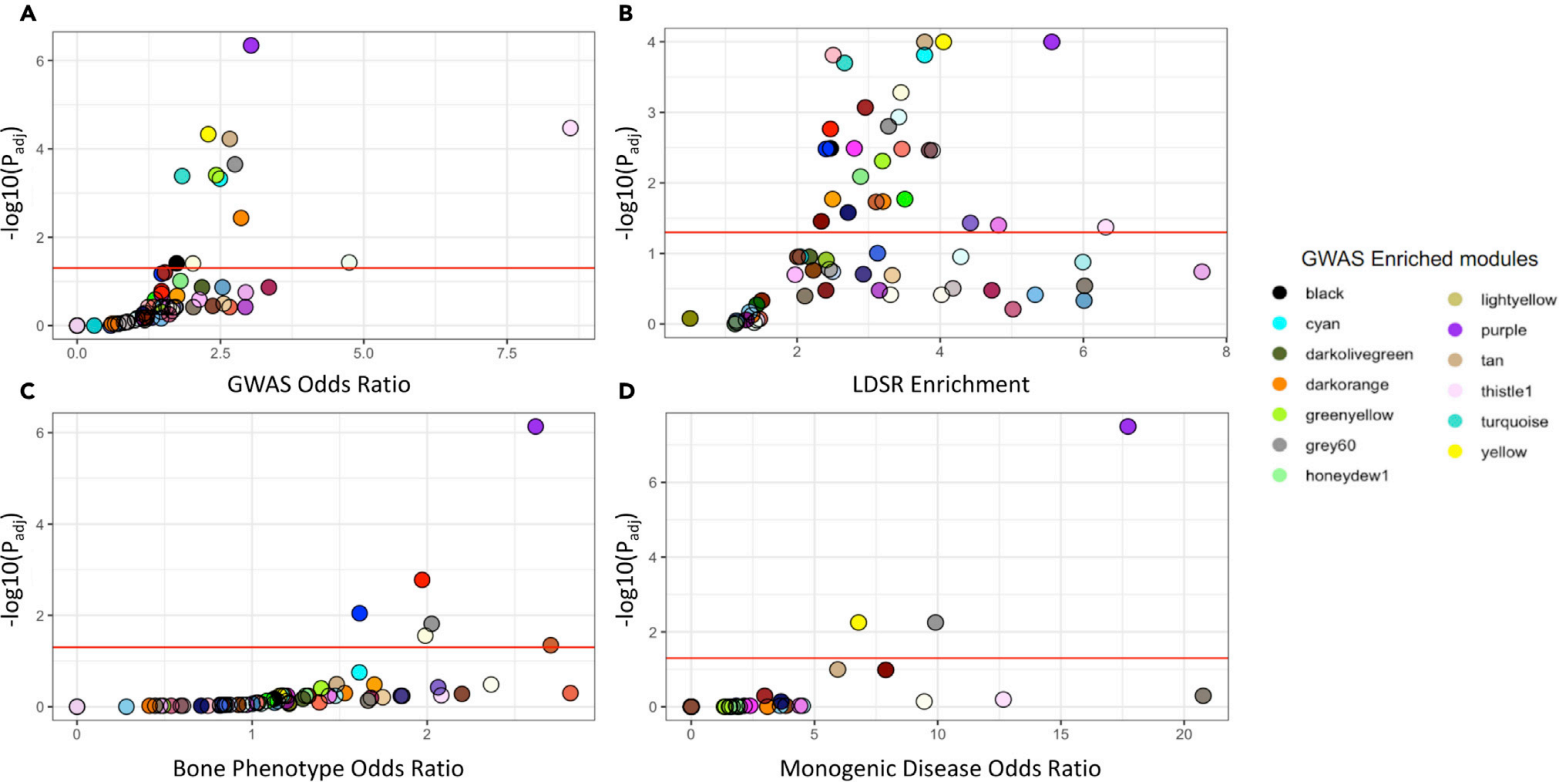
Identifying co-expression modules enriched for genes with “core”-like properties Module enrichment for (A) GWAS genes, (B) GWAS heritability, (C) genes associated with related phenotypes, and (D) genes associated with related monogenic diseases. Adapted from Figure 2 in (Sabik et al., 2020).
  e. In this step of the analysis, the user will need to set their specific
  f. In order to better understand the biological functions that the enrich modules represent, refer back to the results of the gene ontology analysis conducted in section 3. Filter the results of the module you identified in this section to see which gene ontology terms are enriched in your module of interest.

14. Now that a list of genes implicated by GWAS has been produced, we can identify which co-expression modules are enriched for GWAS genes and for the genes previously identified as causal of related monogenic diseases and phenotypes in mice.
  a. First, we define the set of modules in our co-expression network for analysis.
    >colors = unique(moduleColors)
  b. Then, a loop is initiated that begins by defining an object containing annotated information for the genes in each module. Next, a fisher’s exact test is applied to measure the statistical significance of the representation of genes implicated by the GWAS study. In this section a = overlap of module and gene list, b = the size of the gene list minus the overlap, c = the size of the module minus the overlap, and d = the number of genes with quantified expression levels in the RNA-seq experiment.
    >d = as.data.frame(get(paste0(“mod)”,color,“_trx_info”))
    >x = unique(d$external_gene_name) %in% gwas_mouse_genes
    >x = sum(x=TRUE)
    >print(dim(d))
    >print(x)
    >a = x
    >b = (417 – a)
    >c = (dim(d)[1] – a)
    >e = (29255 – c – b – a)
    >assign(paste0(“ft_mod_”, color),
     fisher.test(matrix(c(a,b,c,e), 2, 2, byrow = TRUE),
     alternative = “greater”))
  c. Finally, a table is produced that condenses results for each test, reporting the p-value and the odds ratio, for each module. A p-value adjustment is applied using the Benjamini & Hochberg (FDR) method and the results are returned, sorted by adjusted p-value. We recommend a threshold of FDR < 0.05 to identify enriched modules.
    >gwas_enrichment_results =
     as.data.frame(matrix(nrow = 59, ncol = 3))
    >colnames(gwas_enrichment_results) =
     c(“module_color”, “p_value”, “odds_ratio”)
    >for (i in 1:length(colors)){
     color = colors[i]
     ft = get(paste0(“ft_mod_”, color))
     gwas_enrichment_results[i,1] = color
     gwas_enrichment_results[i,2] = ft$p.value
     gwas_enrichment_results[i,3] = ft$estimate
     }
    >gwas_enrichment_results$p.adj = p.adjust(gwas)enrichment_results$p_value, method = “fdr”, n = length(gwas_enrichment_results$p_value))
    >gwas_enrichment_results %>% arrange(p.adj)
  d. This process can be repeated for each module from the co-expression network and the results can be compared using a scatter plot of the results (Figures 2A, 2C, and 2D), where the enrichment and significance are plotted and each point represents a module. In this case, one module is among the most highly/significantly enriched across all gene sets, which is characteristic of a “core” module. The module that scores highest across all gene sets is a good candidate for a “core” module.

### Step 5: LD score regression

**Timing: 1 h**

Another lens through which we can understand the relationship between the modules in the co-expression network and the GWAS results is through partitioned heritability analysis using LD score regression. This workflow is used to identify modules that are composed of genes related to variants that are enriched for trait heritability in the GWAS study. LD score regression is conducted using the ldsc package, which takes GWAS summary statistics, baseline linkage disequilibrium measurements, and gene sets in which to identify enrichment (Finucane et al., 2015). The partitioned heritability of the SNPs in the regions surrounding the genes in each module is calculated to identify the modules that are most highly enriched for heritability for the trait of interest.

***Note:*** While this package is not wrapped for R, it is accessible in python using the command line, so the code chunks from this section cannot be run in R! The ldsc package wiki on github has a detailed tutorial for the following steps, including links for downloading required files.

15. The first step of LD score regression is to format the GWAS summary statistics for using in the ldsc algorithm, the merge alleles file is provided with the ldsc package

>./munge_sumstats.py \

--out BMD \

--merge-alleles w_hm3.snplist \

--a1-inc \

--sumstats bmd_gwas_sumstats.txt

16. Next, a set of SNPs associated with each module are identified via the genes in each module. This function requires a gene set for each module (as Ensembl gene IDs), a file indicating the coordinates of each Ensembl gene ID (ENSG_coord.txt, provided in the ldsc documentation), the plink file for each chromosome (1000G_plinkfiles/1000G.mac5eur., provided in the ldsc documentation), and a path for the annotation output, indicating the module and the chromosome number. This will need to be run for each chromosome across all modules.

>python ../../src/ldsc/make_annot.py –gene-set-file

violet_module_human_gene_ids.Geneset – gene-coord-file

ENSG_coord.txt –windowsize 100000 – bimfile

 ./100G_plinkfiles/1000G.mac5eur.1.bim –annot-file

 ./violet_annot/violet_module.1.annot.gz

17. Finally, using all of these annotations, run ldsc using the (1) processed summary statistics, (2) the base annotation paths for the modules' annotations, (3) the SNP weights and (4) frequencies for the European 1000 Genomes data that are provided with the ldsc package. The overlap annotations flag was used because transcripts were used to generate the co-expression network, so the gene sets are non-disjoint. Finally, a base name for the output is provided. This command will output a log file, recording the command used to generate the output and a results file. The results file contains a table reporting the proportion of SNPs associated with the gene set, the proportion and standard error of heritability in those SNPs, the enrichment and standard error of the enrichment, and a p-value indicating the statistical significance of the enrichment (Table 1). This table can be filtered to identify the modules that are significantly enriched for trait heritability, and ordered by enrichment to identify the most enriched modules.

**Table 1:** Example output for LD score regression

Module	prop_snps	prop_h2	prop_h2_se	Enrichment	enrich_se	enrich_p	p.adj
purple	0.47	0.26	0.04	5.56	0.90	3.1 **×** 10^−6^	8.8 **×** 10^−5^
Tan	0.41	0.15	0.02	3.78	0.59	3.6 **×** 10^−6^	8.8 **×** 10^−5^

>python ldsc.py

--h2 BMD.sumstats.gz\

--ref-ld-chr antiquewhite4_module., bisque4_module., black_module., blue_modules., ...etc.\

--w-ld-chr ./weights_hm3_no_hls/weights.

--overlap-annot\

--frqfile-chr 1000G.mac5eur.\

--out BMD_all_modules_compare

### Step 6: Gene-level analysis prioritization

**Timing: 30 min**

Once key modules enriched for genes exhibiting core-like properties are identified in sections four and five, the next step is to use these modules as a platform for identifying key genes influencing the phenotype of interest. In sections seven and eight, it may not be feasible to analyze every gene in an enriched module. The topological properties of the nodes within the module can be used to prioritize genes for colocalization and phenstat analysis. In this section, the WGCNA package is used to calculate the module membership score for each gene within a module of interest.

18. Quantitatively, the module membership score is the correlation between the module’s eigengene, which describes the collective expression profile of the group of genes, and each individual gene’s expression pattern. Module membership is highly correlated with intramodular connectivity, and thus, intramodular hub genes tend to have high module membership.

>MMvalue = as.data.frame(cor(norm_exp_by_strain,

 MEs, use = “p”))

>MMPvalue = as.data.frame(corPvaluStudent(

as.matrix(geneModuleMembership), nSamples))

19. Once module membership is calculated for all transcripts, we can annotate all the genes in our modules of interest with their module membership scores, sort the list to identify those genes with the highest module membership scores, and carry those into steps seven and eight.

>module_color = “purple”

>cols = c(paste0(“MM.”, module_color), paste0(“MM.p”, module_color), “MMValue”, “MMPvalue”, “Transcript.ID”)

>mod_MMtable = dplyr::select(MMvalue, one_of(cols)) %>%

 merge(., dplyr::select(MMPvalue, one_of(cols))

>mod_mm_df = merge(mod_MMtable,

 get(paste0(“mod)”, module_color, “_trx_info”)),

 by.x = “Transcript.ID”, by.y = “ensembl_transcript_id”)

>mod_mm_df %>% arrange(desc(MM.purple))

### Step 7: Colocalization analysis

**Timing: 1 h 30 min**

Colocalization analysis is conducted to provide support for hypotheses linking individual genes from core modules with the GWAS phenotype. Colocalization analysis is conducted to evaluate whether two loci share a causal variant. In this instance, the coloc package is implemented to identify relationships between trait-associated GWAS loci and cis expression quantitative trait loci (eQTLs) from Gene-Tissue Expression project (GTEx), however, any relevant eQTL dataset could be used in this step (Giambartolomei et al., 2014, GTEx Consortium et al., 2017). The coloc package evaluates five different hypotheses regarding the relationship between the two associations and returns the probability of each as a value between zero and one: the H0 hypothesis, that neither association is significant, the H1/H2 hypotheses, that only one of the associations is significant, the H3 hypothesis, that both associations are significant but do not share a causal variant, and the H4 hypothesis, that the two associations are significant and share a causal variant. We are interested in identifying the pairs of eQTL and GWAS associations that have a high value of H4 (Table 2).

***Note:*** If no single module is identified as a potential core module in steps 4 and 5, the results may need to be aggregated to create a ranking, see troubleshooting**, Problem 1**.

20. First, for the colocalization analysis, eQTL information from the GTEx consortium will need to be downloaded and filtered (GTEx Consortium et al., 2017). The data are available for download at https://gtexportal.org/home/datasets.

***Note:*** The tarball required for this analysis is 188 Gb in size. It would not be advisable to operate on this data on a laptop, though it may be possible. It is recommended that this file is downloaded directly to a remote server using the command line.

>wget

https://storage.googleapis.com/gtex_analysis_x7/single_tissue_eqtl_data/GTEx_Analysis_v7_eQTL_all_associations.tar.gz

***Note:*** It is possible to use eQTL from any source, however, the below code is designed to work with GTEx data and may need to be modified to work with other eQTL sources.

21. Once the full eQTL data are acquired, the associations for each gene will need to be extracted from each tissue file. This can be accomplished using awk on the command line. These files can all be saved to a subdirectory of the downloaded github repo directory and read in as a part of the loop.

>awk -F “\t” ‘$1 ∼ /ENSG###\ {print}’ .txt | awk -F “\t”

 ‘{ if (($3 >= lower_coord_limit) &&

 ($3 <= upper_coord_limit)) { print } }’ >

 tissue_gene_eqtl_output_file.txt

>gene_files = list.files(“./data/eqtl_data/b4galnt3_snps/”)

22. Furthermore, there are a few additional pieces of information required for input to coloc that have not been used in previous steps of this analysis. The sample size for each underlying GWAS or eQTL study, and the minor allele frequency (MAF) of the variants in each study are required. For the GTEx v7 eQTL studies, a key is provided with the sample sizes for each of these studies. The sample size for the GWAS study should be included in the summary statistics or associated manuscript. Additionally, the MAFs are included as part of the GTEx eQTL association table, and the frequencies of the alleles in the GWAS study are typically reported, however, the MAF is not always the reported frequency, so you may need to convert these frequencies if any are above 0.5. If MAFs are not reported, they can be sourced from the 1000 Genomes project using the LDLink tools.

>gwas_coloc$MAF = ifelse(gwas_coloc$A1FREQ > 0.5,

 (1 – gwas_colos$A1FREQ), (gwas_coloc$A1FREQ))

>tis = read_tsv(“./data/eqtl_data/tissue_key.txt”)

23. With all of this data read in, a loop can now be run that reads in and formats eQTL and GWAS data and runs coloc for each file in the eQTL data folder. The results of the five hypothesis test for each eQTL/GWAS association pait are saved in a dataframe.
  a. First, an empty dataframe is initiated
    >gene_coloc_results = data.frame(matrix(NA, nrow = length(tis$Tissue), ncol = 8))
  b. Next, we initiate a loop that iterates over each eQTL file, reads it in, and formats it with appropriate variant IDs.
    >for (i in 1:length(gene_files)){
     print(i)
     x = gene_files[i]
     y = nchar(gene_files[i]) -23
     tissue = str_sub(x, 1, y)
     z = read_tsv(paste0(“./data/eqtl_data/b4galnt3_snps/”,
     x). col_names = FALSE)
    cols = c(“X2”, “X3”, “X4”, “X5”, “X6”)
    z$variant_id = docall(paste, c(z[cols], sep = “_”))
    z = z[,c(1,14,7,8,9,10,11,12,13)]
    colanmes(z) = c(“gene_id”, “variant_id”,
     “tss_distance”, “ma_samples”, “ma_count”, “maf”,
     “pval_nominal”, “slope”, “slope_se”)
    gene_snp_ids = GTExIdConvert(z$variant_id)
    z = merge(z, gene_snp_ids, by = “variant_id”)
    tissue_n = as.numeric(tis[which(tis$Tissue ==
     tissue),2])
    ...
  c. Finally, the coloc objects for the GWAS and the eQTL are formatted, coloc is run, and the results are written out into a dataframe.

...

gene.coloc = list(pvalues=as.numeric(z$pval_nominal),

 N=as.numeric(tissue_n), type=‘quant’,

 snp=as.character(z$rs_id),

 MAF=as.numeric(z$maf))

gwas.coloc = list(pvalues=as.numeric(gwas_coloc$P),

 N=142487 type=‘quant’,

 snp=as.character(gwas_coloc$SNP),

 MAF=as.numeric(gwas_coloc$MAF))

coloc_x = coloc.abf(gene.coloc, gwas.coloc)

gene_coloc_results[i,] =

 c(tissue,z$gene_id[1],

 coloc_x$summary[1],

 coloc_x$summary[2],

 coloc_x$summary[3],

 coloc_x$summary[4],

 coloc_x$summary[5],

 coloc_x$summary[6])

}

**Table 2:** Example output for colocalization analysis using the coloc package

Tissue	Gene	Nsnps	PP.H0.abf	PP.H1.abf	PP.H2.abf	PP.H3.abf	PP.H4.abf
Adipose_Subcutaneous	ENSG00000139044	858	2 **×**10^−12^	3 **×**10^−6^	8 **×**10^−7^	.999	3 **×**10^−6^
Adipose_Visceral_ Omentum	ENSG00000139044	858	5 **×**10^−14^	3 **×**10^−6^	2 **×**10^−8^	.999	6**×**10^−8^

The resulting table will report the tissue (“tissue”), the gene ID (“gene”), number of SNPs included in the coloc analysis (“nsnps”), and the posterior probability of the four hypotheses assessed by coloc (“PP.H0.abf” = no significant association, “PP.H1.abf” = only the eQTL association was significant, “PP.H2.abf” = only the GWAS association was significant, “PP.H3.abf” = the associations are both significant and likely have independent genetic drivers, “PP.H4.abf” = the associations are both significant and likely share a common genetic driver).

**Figure 3:**
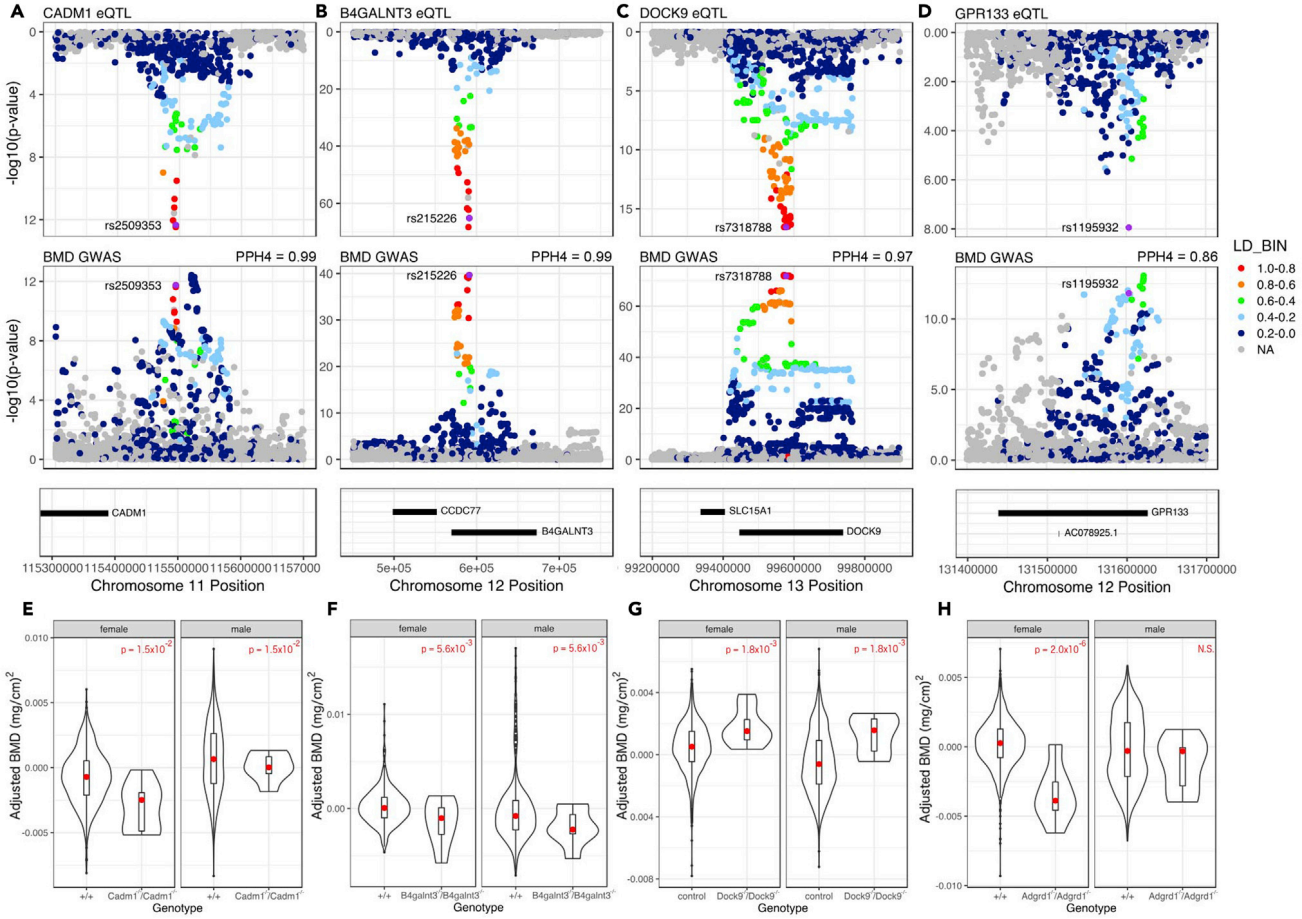
Identifying core genes from a core module (A–D) represent the results of positive colocalization analyses, where there was a sufficiently high PP H4 to indicate that the BMD GWAS signal and the GTEx eQTL for the given gene shared a common genetic driver. (E–H) represent significant differences in a phenotype (bone mineral density) in knockout mice from the IMPC database, as analyzed by PhenStat. Adapted from Figure 5 in (Sabik et al., 2020).

***Optional:*** These results can also be visualized with the RACER package (Sabik and Farber, 2018)(Figures 3A–3D). A detailed vignette for using the RACER package can be found here: https://oliviasabik.github.io/RACERweb/articles/IntroToRACER.html.

### Step 8: PhenStat analysis

**Timing: 1–2 hrs**

While the colocalization analysis provides evidence supporting a relationship between network identified genes and a trait of interest, a causal relationship can only be demonstrated through controlled perturbation of a target and direct measurement of the phenotype of interest. The hypotheses generated by the above steps can lead to a novel set of experiments, however, databases of experimental perturbations and measured phenotypes can be mined for evidence supporting a causal relationship between a gene and a phenotype of interest. For example, the International Mouse Phenotyping Consortium has a database of phenotypes measured in 7022 strains of knockout mice as of release 12.0 (Koscielny et al., 2014).

24. Identify genes from your core module, perhaps those with eQTL that colocalize with the GWAS of interest that are represented in the IMPC database.
  a. From their home page (https://www.mousephenotype.org/), search by gene name for potential causal genes.
  b. Navigate to a gene of interest to see what phenotypes are available, for example the page for the gene B4galnt3. (https://www.mousephenotype.org/data/genes/MGI:3041155).
  c. IMPC reports which genotype/phenotype relationships are significant above a multiple testing threshold for all genes; however, they make all data available for testing a single hypothesis.
  d. The data may be downloaded by clicking on a relevant phenotype icon (https://www.mousephenotype.org/data/charts?accession=MGI:3041155&allele_accession_id=MGI:4434237&pipeline_stable_id=ESLIM_001&procedure_stable_id=ESLIM_005_001&parameter_stable_id=ESLIM_005_001_004&zygosity=homozygote&phenotyping_center=ICS), scrolling to the “Access the results programmatically” section and clicking to download the “PhenStat-ready raw experiment data”.
  e. This data can be loaded in R and the analysis can be run using the PhenStat package (Kurbatova et al., 2015). First, the data are read in:
    >url = ‘htps://www.mousephenotype.org/data/exportraw? phenotyping_center=JAX&parameter_stable_id=IMPC_DXA_004_001&allele_accession_id=MGI:5804021&strain=MGI:3056279&pipeline_stable_id=JAX_001&&zygosity=homozygote&’
    >dataset1 = data.table::fread(url)
    >dataset1 = as.data.frame(dataset1)
    >dataset1 = dataset1[,c(15,16,21,26,28)]
  f. Next, a PhenList object is created for Phenstat
    >test = PhenList(dataset1,
     testGenotype=”JR29804”,
     dataset.colname.batch = “Assay.Date”,
     dataset.value.male = “male”,
     dataset.value.female = “female”,
     dataset.clean = TRUE,
     outputMessages = TRUE)
  g. Next, use the testDataset function to test for differences in a dependent variable, here "Value". The program will choose whether to keep specific model effects, and report if it corrects for batch, weight, and sex, or an interaction term. Additionally, it does not detect a difference in variance between the genotype groups.
    >results_MM_bmd = testDataset(test, depVariable = “Value”)
  h. Use the summaryOutput function to view the results of the statistical test for comparing the knockout phenotype against the control.
    >summaryOutput(results_MM_bmd)
  i. Finally, create a boxplot of the differences in the value between genotypes for both sexes using the boxplotSexGenotype function, or the boxplotSexGenotypeBatchAdjusted if the effect of batch was significant.
    >boxplotSexGenotype(test,
     depVariable = “Value”,
     graphingName = ‘Bone Mineral Density”,
     outputMessages = T)
    >PhenStat::boxplotSexGenotypeCatchAdjusted(test,
     depVariable = “Value”)
  j. The adjusted variables can also be extracted, using the getColumnWeightBatchAdjusted function, and plotted using a different package, for example, ggplot2 (Figures 3E–3H).

## Expected outcomes

The expectation of this analytical pipeline is that the results of the module enrichment analyses will identify one, or a small set of modules that will harbor significant enrichment for genes that exhibit core-like properties. As an example, in the original implementation of this pipeline to study bone mineral density, one group of co-expressed genes, the purple module, was the most highly enriched for genes associated with a number of core-like properties. The purple module was the most highly enriched (1) for genes associated with genes that, when knocked out in mice, exhibited an effect on bone mineral density, (2) for genes related to osteoblast-related monogenic diseases, including osteogenesis imperfecta, hyperostosis, and osteosclerosis, and (3) for genes implicated by GWAS and GWAS heritability (Figure 2). However, the expectation that there will be a clear subset of modules enriched across all core-like properties that may not hold, see troubleshooting Problem 1.

## Limitations

This protocol relies heavily on publicly available data, which can be both a benefit and an obstacle. It may be the case that there is useful data in each of the resources listed here, however, it is likely that only a subset of applicable data is available for the phenotype in question. In this case, specific, bespoke experiments may need to be performed to demonstrate the hypotheses in question. Additionally, this protocol requires knowledge of the R language and running tools on the command line, including python packages and awk. While the vignette on Github was created to help novice users by providing example data and examples of each step of this protocol, newer users may still need to learn some of the skills underlying the protocol to execute it fully.

## Troubleshooting

### Problem 1

In the before you begin section, in “Curating lists of known disease and phenotype genes” , you may find that the gene lists you identify for your trait of interest from the databases mentioned are not sufficiently large to identify any enriched modules

### Potential solution

While we list a couple of options for sources of disease and phenotype associated genes, the genes relevant to your analysis may not be as heavily represented and curated gene lists may need to be manually curated from the literature.

### Problem 2

In steps three and four, gene ontology analysis and enrichment analysis, it may be the case that too many of the modules from the co-expression network are too small to be significantly enriched for GO terms, GWAS genes, or genes associated with monogenic diseases.

### Potential solution

This can be addressed by increasing the minModuleSize parameter in the network construction command in step two. This will result in a small number of larger modules, which may elide some nuances between smaller modules, but will allow for more successful enrichment analyses.

### Problem 3

In steps three through five, gene ontology, enrichment analysis, and LD score regression, you may find that no single module is remarkable across the GWAS, phenotype, and monogenic disease-related gene enrichment analyses and partitioned heritability analysis.

### Potential solution

If this is the case, optimizing over the module ranks for each enrichment will lead to a prioritized list of modules, which can be carried into the steps seven and eight, colocalization and Phenstat analysis.

### Problem 4

In step seven, colocalization analysis, there are a limited number of tissues in GTEx. It is possible that none of the tissues present in GTEx are relevant to the GWAS phenotype at the center of the analysis.

### Potential solution

In the protocol, the solution implemented is to analyze all available tissues, which can still surface pan-tissue effects. Another option is to use an eQTL dataset more fit for purpose. Recently, the eQTL Catalog has been made available by the EMBL-EBI (Kerimov et al., 2020).

### Problem 5

It is assumed that there is a viable surrogate phenotype in the IMPC for step eight, however there is a limited number of phenotypes available from the IMPC and there may not be one that is fit for purpose.

### Potential solution

If there is not a suitable surrogate phenotype in the IMPC database, additional experiments may be required to demonstrate a causal relationship between the gene and phenotype of interest.

## Resource availability

### Lead contact

Further information and requests for resources and reagents should be directed to and will be fulfilled by the lead contact, Charles Farber (crf2s@virginia.edu).

### Materials availability

This study did not generate new unique reagents.

## Data Availability

The accession number for the RNA sequencing data used as an example in this paper is GEO: GSE134081. The code is available on github at: https://github.com/Farber-Lab/STAR_protocols_core_modules.
